# Somatic LINE-1 promoter acquisition drives oncogenic *FOXR2* activation in pediatric brain tumor

**DOI:** 10.1007/s00401-022-02420-9

**Published:** 2022-04-11

**Authors:** Diane A. Flasch, Xiaolong Chen, Bensheng Ju, Xiaoyu Li, James Dalton, Heather L. Mulder, John Easton, Lu Wang, Suzanne J. Baker, Jason Chiang, Jinghui Zhang

**Affiliations:** 1 Department of Computational Biology, St. Jude Children’s Research Hospital, Memphis, TN USA; 2 Department of Pathology, St. Jude Children’s Research Hospital, Memphis, TN USA; 3 Department of Developmental Neurobiology, St. Jude Children’s Research Hospital, Memphis, TN USA

Long Interspersed Element-1 (LINE-1 or L1) is the only autonomously active human retrotransposable element shown to mobilize in cancers, which can disrupt normal gene function or regulation [6]. However, L1 regulatory elements have not been implicated in human tumorigenesis.

We identified an infant high-grade glioma (HGG, Fig. 1a) showing DNA methylation profiles (Fig. 1b) and *FOXR2* overexpression (Fig. 1c) characteristic of *FOXR2*-activated CNS neuroblastoma (NBL) [1]. However, histology review confirmed typical HGG findings—infiltrating astrocytic tumor cells demonstrated strong and diffuse GFAP expression and were negative for synaptophysin. This suggests that aberrant *FOXR2* activation may have driven tumorigenesis and the observed methylome profile.

**Fig. 1:**
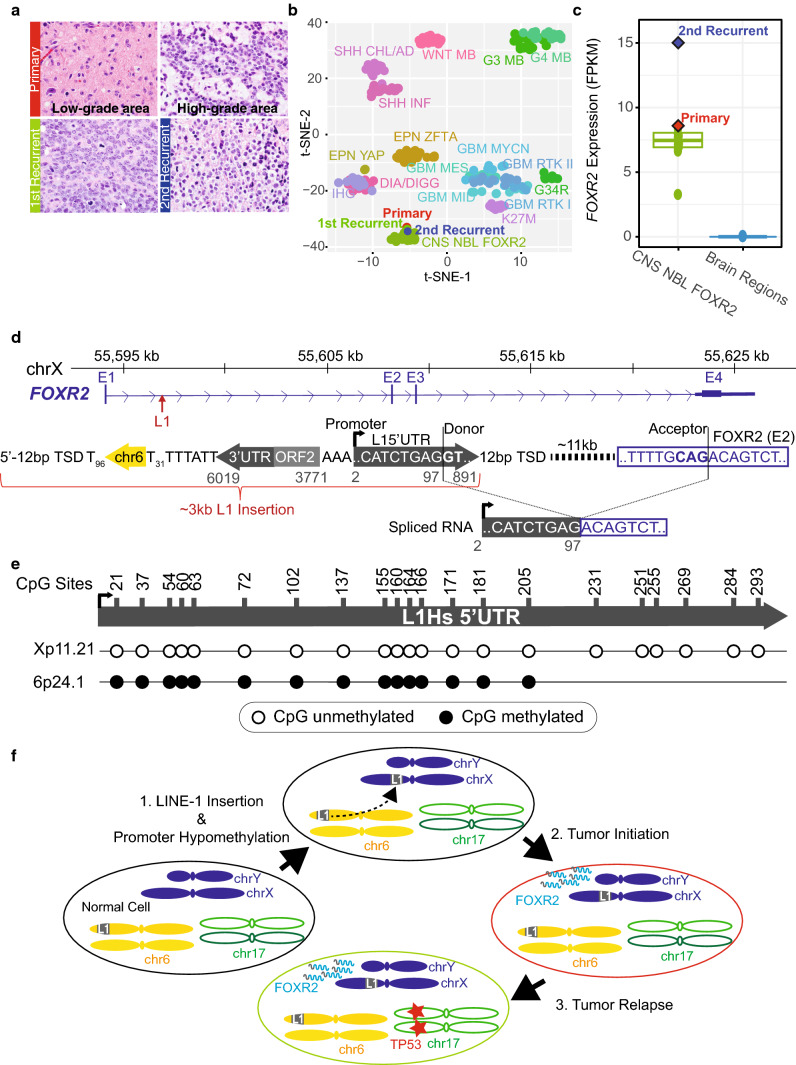
Somatic L1 promoter donation drives oncogenic *FOXR2* overexpression. **a** Histology of serial tumor samples. **b** t-SNE plot comparing genomic DNA methylation profiles of the primary and recurrent tumors to 249 pediatric CNS tumors of 17 types (see Supplementary text, online resource for additional abbreviations).** c** *FOXR2* expression of CNS NBL FOXR2 overlayed by the primary and 2nd recurrent tumors as compared to non-diseased multiple brain regions profiled by GTeX. **d** Top, schematic of PacBio sequenced L1 insertion with a chr6 transduction sequence (yellow arrow) and flanked by target-site duplication sequence (TSD). Gray numbers match nucleotides of L1.3 consensus sequence. Below, observed tumor transcripts involve L1 splice donor to canonical *FOXR2* splice acceptor. **e** Methylation status of CpG sites (nucleotide numbers) in the L1 5’UTR at the retrotransposed *FOXR2* locus (Xp11.21) and the source element (6p24.1) observed in over 90% of bisulfite sequencing reads. **f** Model of oncogenic activation with chr6 L1 source element (gray ‘L1’ box) insertion in chrX upstream of *FOXR2*, inducing oncogenic overexpression of *FOXR2* (gray and blue squiggles), driving the primary tumor. Recurrent tumor formed, acquiring a *TP53* R175H variant (red star)

The tumor’s whole genome sequencing (WGS) data revealed a cluster of soft-clipped (SC) reads containing sub-regions unmapped to the reference genome located within intron 1 of *FOXR2*. The reads contained a poly-A or L1 5’UTR sequence, indicating an L1 insertion event (Supplementary Fig. 1a, online resource). PCR amplification of the genomic sequence revealed a ~ 3 kb somatic insertion (Supplementary Fig. 1b, online resource). Targeted PacBio sequencing identified a 5’ inverted L1 insertion with a nearly intact L1 5’UTR, which contains an RNA pol-II promoter in the same orientation as *FOXR2* but inverted with respect to the remaining truncated L1 sequence, where a partial L1 open reading frame (ORF2) was present, followed by the L1 3’UTR, a 31 bp poly-A tail, a 29 bp transduction sequence, and a 96 bp poly-A tail (Fig. 1d). The insertion site was flanked by a target-site duplication (TSD; 5’-GTTGATATCTTT). The transduction sequence enabled us to trace the full-length 6p24.1 L1 as the source element responsible for the somatic insertion (Supplementary Fig. 1c, online resource) [2, 4], which was also confirmed by shared L1 sequence variants between the 6p24.1 L1 and the *FOXR2* L1 (Supplementary Table 1, online resource).

RNA-seq data indicated “donation” of the L1 promoter initiated *FOXR2* transcription as we identified a chimeric L1*/FOXR2* transcript spanning the first 97 bp of L1 5’UTR from a known L1 splice donor site to the acceptor site of exon 2 of a non-canonical *FOXR2* isoform (Fig. 1d and Supplementary Fig. 2b, online resource) [3]. There was no expression of *FOXR2* exon 1 nor splice junction reads upstream the L1 insertion (Supplementary Fig. 2a, online resource). To further confirm promoter activity of the *FOXR2* L1, we performed bisulfite sequencing on its 5’UTR. We observed hypomethylation of all CpG sites profiled, while the source 6p24.1 L1 5’UTR remained hypermethylated (i.e., inactive) (Fig. 1e). These results support an active L1 promoter driving aberrant *FOXR2* transcription in the tumor.

Molecular profiling of serial tumor samples projected the temporal order of mutation acquisition as follows (Fig. 1f): a somatic L1 insertion at the *FOXR2* locus led to aberrant oncogenic *FOXR2* expression and chimeric L1/*FOXR2* transcripts. The insertion was an early tumor-initiating event, as it was the only driver present at diagnosis and, as a founder mutation, persisted through tumor recurrence. While wild-type p53 expression was confirmed in the primary tumor, a clonal *TP53* R175H mutation with loss of heterozygosity was acquired in recurrent tumors (Supplementary Fig. 3, online resource).

Our study presents the first example of L1 promoter “donation” as a novel cancer-initiating mechanism, as compared to previously reported L1-mediated disruption of tumor suppressors or oncogene repressors [6]. We screened an additional 183 pediatric HGG samples and 22 CNS tumors [7] and did not observe another L1/*FOXR2* fusion, likely due to low L1 activity in CNS tumors [6]. Nevertheless, the findings made in the index HGG broaden oncogenic L1 retrotransposition mechanisms, providing a new direction for investigating genomic drivers in non-coding regions. Optimal treatment strategies for this hybrid histological HGG and molecular CNS NBL FOXR2 tumor demand further investigation which may involve assessing the functional impact of FOXR2 activation, known to stabilize cMYC [5], on global methylome changes in neural progenitor cells.

## Supplementary Information

Below is the link to the electronic supplementary material.

Supplementary file1 (XLSX 58 KB)

Supplementary file2 (PDF 7561 KB)

## References

[1] WHO Classification of Tumours Editorial Board (2021) Central nervous system tumours [Internet]. Lyon (France): International Agency for Research on Cancer (WHO classification of tumours series, 5th ed; vol 6). Available from: https://tumourclassification.iarc.who.int/chapters/45. Accessed 9 Mar 2022

[2] Beck CR, Collier P, Macfarlane C, Malig M, Kidd JM, Eichler EE (2010). LINE-1 retrotransposition activity in human genomes. Cell.

[3] Belancio VP, Hedges DJ, Deininger P (2006). LINE-1 RNA splicing and influences on mammalian gene expression. Nucleic Acids Res.

[4] Kidd JM, Cooper GM, Donahue WF, Hayden HS, Sampas N, Graves T (2008). Mapping and sequencing of structural variation from eight human genomes. Nature.

[5] Li X, Wang W, Xi Y, Gao M, Tran M, Aziz KE (2016). FOXR2 interacts with MYC to promote its transcriptional activities and tumorigenesis. Cell Rep.

[6] Scott EC, Devine SE (2017). The role of somatic L1 retrotransposition in human cancers. Viruses.

[7] Sturm D, Orr BA, Toprak UH, Hovestadt V, Jones DTW, Capper D (2016). New brain tumor entities emerge from molecular classification of CNS-PNETs. Cell.

