# Supplementary material for: Somatic LINE-1 promoter acquisition drives oncogenic FOXR2 activation in pediatric brain tumor
Source: Acta Neuropathol. 2022 Apr 11;143(5):605–7. doi: 10.1007/s00401-022-02420-9 (PMC9038871; doi:10.1007/s00401-022-02420-9)
Supplement: Supplementary file 2 — Supplementary file2 (PDF 7561 KB) [file 401_2022_2420_MOESM2_ESM.pdf]

Supplementary Information for

Somatic LINE-1 Promoter Acquisition Drives Oncogenic *FOXR2* Activation in Pediatric Brain Tumor

Diane A. Flasch, Xiaolong Chen, Bensheng Ju, Xiaoyu Li, James Dalton, Heather L. Mulder, John Easton, Lu Wang, Suzanne J. Baker, Jason Chiang, Jinghui Zhang

Correspondence to: Jason.Chiang@stjude.org; Jinghui.Zhang@stjude.org

This file includes:

Supplementary Figures 1 to 3
Supplementary Tables 1 to 5

Abbreviations for Fig. 1b:
CNS tumors of 17 entities, Abbreviations: CHL/AD: child/adult; DIA/DIGG: Desmoplastic infantile astrocytoma/ganglioglioma; EPN: Ependymoma; CNS NBL FOXR2: *FOXR2*-activated CNS neuroblastoma; G3: Group 3; G34R: Histone H3 G34R-mutant diffuse hemispheric glioma; G4: Group 4; GBM: Glioblastoma; IHG: Infant-type hemispheric glioma; INF: Infant; K27M: Histone H3 K27M-mutant diffuse midline glioma; MB: Medulloblastoma; MES: Subclass mesenchymal; MID: Subclass midline; MYCN: Subclass MYCN; RTKI/II: Subclass RTKI/II; SHH: SHH-activated medulloblastoma; WNT: WNT-activated; YAP: *YAP1*-fused; ZFTA: *ZFTA*-fused

Additional Results and Discussion:

Histopathological and molecular characterization of the index tumor samples
The second recurrent tumor sample of our index patient was subjected to our clinical cancer genomic profiling by three-platform sequencing of whole-genome, whole-exome, and

transcriptome [31], and harbored two additional somatic pathogenic variants: a homozygous clonal *TP53* R175H mutation and a subclonal *PDGFRA* amplification (Supplementary Fig. 3). The patient was initially diagnosed with HGG at 11 months of age, with a tumor that showed both low- and high-grade histology and a wild-type expression pattern of p53 as demonstrated by immunohistochemistry (Supplementary Fig. 3e). After receiving chemotherapy treatment, the high-grade glioma relapsed a year later and contained a clonal *TP53* mutation, a common feature of pediatric HGG but exceptionally rare for CNS NBL FOXR2. After irradiation treatment, a year later, the tumor recurred for a second time with an even stronger expression of mutant p53 (Supplementary Fig. 3e) and a subclonal copy gain of *PDGFRA* identified by methylation array (Supplementary Fig. 3c), both are common in pediatric HGG but not CNS NBL FOXR2. The subclonal *PDGFRA* amplification, potentially associated with conferring therapeutic resistance, was observed only in the second recurrent tumor. These findings were corroborated by both WGS and FISH (Supplementary Fig. 3d). Interestingly, the DNA methylation profiles of the primary and recurrent tumors (1st and 2nd) all clustered with reference tumors of CNS *FOXR2*-altered NBL, suggesting a common lineage related to *FOXR2* activation (Fig. 1b). The results of DKFZ methylation classifier (molecularneuropathology.org) also support this finding (calibrated scores 0.23804, 0.72338, and 0.99994 for CNS NBL FOXR2 for the primary and 1st and 2nd recurrent tumors, respectively). We were able to perform additional whole-exome and RNA-seq on the primary tumor sample acquired at diagnosis. Exome data showed the absence of any pathogenic coding variants at diagnosis, consistent with the wild-type *TP53* status by immunohistochemistry. More importantly, RNA-seq data displayed transcription of the non-canonical *FOXR2* isoform as well as the *L1/FOXR2* fusion transcript (Supplementary Fig. 2a),

confirming the presence of the somatic L1 insertion and activation of *FOXR2* expression driven from the inserted L1 promoter sequence at diagnosis.

Prevalence of somatic L1 insertion in pediatric high-grade gliomas

While *de novo* somatic L1 insertions have been studied extensively in adult cancers [16, 33, 34, 38], and epithelial cancers show higher L1 mobilization and subsequent *de novo* insertion rates as compared to brain and blood cancers [1, 20], yet the activity levels of these elements in pediatric cancers have not been well studied. Following the discovery made on the index HGG, we performed a screening of somatic L1 insertions on 87 HGG paired tumor/normal WGS data sets by filtering somatic insertions identified by the structural variant caller Manta [4] and by running the mobile element detector MELT [14] and TraFiC-mem [29](Supplementary Methods). We identified three additional pediatric tumor samples, each showing evidence of a single somatic L1 insertion, albeit intergenic events (Supplementary Methods, Supplementary Table 3), consistent with the low L1 insertion rate observed in CNS tumors previously [1, 20].

Prevalence of L1 promoter donation in pediatric brain tumors

To determine the prevalence of transcript activation by this L1 "promoter donation" mechanism, we analyzed tumor RNA-seq from an additional 182 pediatric HGG samples and 22 previously published CNS tumors in a study that reported *FOXR2*-activated CNS neuroblastoma as a new brain tumor entity [36]. We screened for the presence of fusion transcripts involving splicing of an L1 97 SD sequence to a genomic location. While we identified several transcripts matching this profile, most were likely caused 'spuriously' by nearby known polymorphic L1s or older L1 lineage insertions as has been previously described [2], or exhibited characteristics of 'exonization'[30, 32, 39] with additional upstream reads also displaying splicing to L1 sequence (Supplementary Table 2 and Supplementary Fig. 2c).

Promoter donation as a new L1-mediated oncogenic mechanism

Repurposing an active promoter of a retrotransposed L1 for oncogene activation is a new mechanism in human cancers unveiled by the in-depth genomic, transcriptomic, and epigenomic analysis presented in this study. The transduction sequence observed in the *FOXR2* L1 somatic insertion indicates the event was most likely a retrotransposition event of the 6p24.1 L1 which was identified as one of the top five "hot" L1 source elements that gave rise to half of all somatic L1 insertion events in adult cancers in a recent pan-cancer study [29]. The transduction occurred with an L1 mRNA transcript that bypassed the canonical L1 polyadenylation signal (5'-AATAAA) and terminated at an alternative downstream polyadenylation signal (5'-AATATA). Furthermore, the presence of the poly-A tail, TSDs and an endonuclease (EN)-cut site (5'-TCTTT/AT) indicates that the *FOXR2* L1 somatic insertion event occurred by canonical L1 EN-mediated retrotransposition.

In contrast, previously reported driver somatic L1 insertion events in adult cancers revealed mutagenesis by disruption, i) either disruption of a tumor suppressor gene (*i.e., APC* in colorectal cancer [25, 34] or *PTEN* in uterine cancer [16]), or ii) disruption of a repressive regulatory element allowing expression of an oncogene (*i.e., ST18* in liver cancer [35]), or iii) creation of an intronic novel *cis*-enhancer driving expression of a putative oncogene (*i.e., STC1* in ovarian cancer [27]). Although a previous pan-cancer analysis of transposable elements cited several examples of oncogene overexpression accompanied by nearby somatic L1 insertions, it should be noted that all these nearby L1 insertions were 5' promoter-truncated (*e.g.,* lacking 5'UTRs), thus ineligible for promoter "donation" [29]. Importantly, through the analysis of serial samples, we show that the somatic L1 insertion was the initiation event present in the

primary tumor, while the other pathogenic variants, *e.g.*, *TP53* mutation and *PDGFRA* amplification, were detected only at tumor recurrence. Thus, promoter "donation" by somatic L1 retrotransposition represents a new mechanism for oncogenesis in human cancers and potentially for aberrant activation of genes involved in other human diseases. It should be noted that popular somatic L1 insertion detection tools, such as MELT [14] and TraFiC-mem [29], the latter of which was previously used for large-scale pan-cancer analysis of WGS data, were unable to identify the somatic *FOXR2* L1 insertion identified in this study [14, 29], suggesting that these events may be underrepresented in the current somatic L1 insertion datasets and may be contributing to more pathogenic alterations than currently appreciated.

Materials and Methods:

Data sets

All WGS and RNA-seq data were downloaded from the St. Jude Cloud (www.stjude.cloud) [24]. WGS were mapped to GRCh38 (GRCh38_no_alt) with BWA-MEM [22] alignment of RNA-seq were mapped to GRCh38 (GRCh38_no_alt) by STAR [9]. Specifically, the index tumor is a 2nd recurrent high grade glioma (SJHGG030242_D1) which, along with its matching normal (SJHGG030242_G1), was DNA and RNA extracted and profiled by the three-platform WGS, exome, and transcriptome sequencing employed by the Genome for Kids study and patient consent was given as previously stated [26]. HGG RNA-seq (n=182) and paired tumor-normal WGS (n=87) used for recurrence screening was profiled by the Pediatric Cancer Genome Project [11], Genome for Kids [26], or Real-Time Clinical Genomics (Supplementary Table 5). We also downloaded and analyzed the RNA-seq data of 22 previously published CNS tumors (EGAD00001001927) which were used for identifying a new brain tumor entity, *FOXR2*-

activated CNS neuroblastoma [36]. Additionally, the authors of the publication provided us with FPKM values from Affymetrix array FOXR2-activated CNS neuroblastomas (GEO accession: GSE73038) [36]. We downloaded and processed 453 GTeX V7 normal brain tissue samples (99 Anterior cingulate cortex (BA24), 130 Cortex, 117 Frontal Cortex (BA9), and 107 Hippocampus) and compared the expression of *FOXR2* with FPKM per gene calculated in RSEM [21] based on Gencode v31 annotation. The L1 consensus sequence used for analysis comparisons was that of L1.3 described and published previously [10].

RNA isolation and sequencing on SJHGG030242 primary tumor

A PureLink FFPE Total RNA Isolation Kit (Thermo Fisher Scientific) was used for total RNA extraction from formalin-fixed paraffin-embedded (FFPE) tumor tissue as previously described [6, 8, 15]. Purified RNA was quantified on a Qubit 3 Fluorometer (Thermo Fisher Scientific) using Qubit™ RNA BR Assay Kit (Thermo Fisher Scientific). Total RNA sequencing was performed using the Illumina TruSeq Stranded Total RNA protocol with at least 500 ng of total RNA. The quality of the starting materials was checked with the RNA 6000 Nano Assay on a 2100 Bioanalyzer (Agilent) or the RNA Pico Sensitivity Assay on a LabChip GX Touch (PerkinElmer). Libraries were prepared using the TruSeq Stranded Total RNA Sample Prep Kit (Illumina), followed by library quantification through qPCR using Quant-iT™ PicoGreen dsDNA Assay Kits (Thermo Fisher Scientific) or KAPA Library Quantification Kits for Illumina platforms (KAPA Biosystems), and through low pass sequencing on a MiSeq Nano v2 (Illumina). All sequencing data were generated after 100 cycles of paired-end runs on an Illumina HiSeq 2500 or HiSeq 4000.

DNA isolation and whole exome sequencing on SJHGG030242 primary tumor

Genomic DNA was extracted from formalin-fixed paraffin-embedded (FFPE) tumor tissue using a QIAamp DNA FFPE Tissue Kit (Qiagen) as previously described [15]. At least 250 ng of genomic DNA was used for each sample. DNA quality was assessed on a 4200 TapeStation (Agilent). Genomic DNA libraries were generated using the SureSelectXT Kit (Agilent Technologies), followed by exon enrichment using the SureSelectXT Human All Exon V7 bait set (Agilent Technologies). The resulting exon-enriched libraries were subjected to paired-end, 100-cycle sequencing performed on a HiSeq 4000 (Illumina).

Methylation array analysis

Genomic DNA extracted from formalin-fixed paraffin-embedded (FFPE) tumor samples was used for genome-wide methylation profiling and CNV analysis by the Illumina Infinium MethylationEPIC platform as previously described [5-8, 15, 17, 23]. At least 250 ng of genomic DNA was used for each sample. For comparison, publicly available well-characterized reference methylation profiles of brain tumors were downloaded from the Genomic Data Commons Data Portal (https://portal.gdc.cancer.gov/). Analysis of methylation profiles, including normalization, filtering, t-distributed stochastic neighbor embedding (t-SNE), and CNV analysis was performed in R v4.1.0 using ChAMP v2.22.0 [37], minfi v1.38.0 [13], limma v3.48.0 [28], conumee v1.26.0, and Rtsne v0.15 (with theta = 0.0) packages in Bioconductor v3.13 (http://bioconductor.org/) as previously described [5-8, 15, 17, 23]. Raw signal intensities were normalized by performing background correction and a dye-bias correction for both color channels with the functional normalization method. The following filtering criteria were applied: removal of probes targeting the X and Y chromosomes; removal of probes containing single-nucleotide polymorphisms; and removal of probes not mapping uniquely to the human reference

genome (hg19), allowing for one mismatch, after removal of poor-quality ($P > 0.01$) and failed probes. Beta values of the 5000 most variable CpG sites were derived for t-SNE analysis.

Histopathology review and immunohistochemistry

Histopathology was centrally reviewed by a neuropathologist specializing in pediatric CNS tumors (J.C.). Standard hematoxylin and eosin histopathologic preparations from each tumor were supplemented by immunohistochemistry on 5-µm formalin-fixed, paraffin-embedded (FFPE) tissue sections. Monoclonal anti-p53 antibody (Zeta Corp, Z2029M, clone DO-7, diluted 1:200) was used to identify the nuclear accumulation of mutant p53. Staining was performed on a Ventana Benchmark Ultra automated stainer with 20-minute CC1 pretreatment and 32-minute incubation with the antibody at 37 °C. IVIEW DAB detection kit was used to visualize the staining results.

Interphase fluorescence *in situ* hybridization

Amplification of *PDGFRA* (4q12) was detected by interphase fluorescence *in situ* hybridization in a Clinical Laboratory Improvement Amendments (CLIA)-certified laboratory in St. Jude Children's Research Hospital with probes developed in-house using the following BAC clones: *PDGFRA* (RP11-231C18 + RP11-601I15) with 4p12 control (CTD-2057N12 + CTD-2588A19), as previously described [15].

Targeted Amplification and PacBio Sequencing

Targeted amplification of the region on chromosome X containing the putative L1 insertion was performed using extracted DNA from the 2nd recurrent tumor (SJHGG030242_D1) and paired normal (SJHGG030242_G1) with Kapa HiFi HotStart Ready Mix KR3070, 300 nM final concentration of the flanking primers (Line_ChrX-For/Line_ChrX-Rev), and 25 ng of genomic

DNA in a 25 ul reaction volume. After the initial denaturation at 95˚C for 5 minutes, PCR was completed with 35 cycles of amplification consisting of a 20 second denaturation at 98˚C, 15 second annealing at 62˚C, and an 8 minute extension at 72˚C. A final extension of 10 minutes was performed prior to a 4˚C hold step. A ~3,800 bp PCR fragment amplified from the 2nd recurrent tumor which contains the putative LINE-1 insertion and a 423 bp fragment from the matching paired normal (SJHGG030242_G1) were used to prepare the PacBio sequencing libraries following the "Procedure & Checklist-Preparing SMRTbell® Libraries using PacBio® Barcoded Overhang Adaptors for Multiplexing Amplicons". The libraries were loaded at 6 pM and run on the PacBio Sequel system using 1 1M v3 LR SMRT Cell.

The PacBio generated circular consensus sequence (CCS) fastq files were then visually analyzed and compared to the L1.3 consensus sequence with SerialCloner v2.6.1 to determine the details of the inserted sequence. Sequence quality generated by PacBio using CCS reads is of high quality based on previously published data showing the ability of PacBio to successfully sequence through L1 poly-A sequence [12].

Bisulfite Conversion, PCR amplification, and Illumina MiSeq Sequencing

Non-FFPE tissue DNA samples in this study were bisulfite converted using the Zymo EZ DNA Methylation-Gold Kit D5006, with 500 ng of DNA under standard protocol conditions. Amplification of the chr6p24.1 source element was performed using the previously published primers in Tubio et al., 6p24.1 F (chr6:13190940-13190966; hg19) and 6p24.1 R (L1Hs:206-229)(Supplementary Table 4, chr6_tubio_for_bs_conv and chr6_tubio_rev_bs_conv) [38]. The 25ul PCR reaction tube (Micron Low bind 0.2 ml PCR tube) contained Kapa HiFi HotStart Uracil + Ready Mix KK2801, Gibco Ultra Pure H_2O (DNAse/RNAse free), 300 nM each

chr6_tubio_for_bs_conv and chr6_tubio_rev_bs_conv PCR primers. All PCR reactions were performed in a BioRad C1000 thermocycler with an initial denaturation for 3 minutes at 95˚C, followed by 38 cycles of a 20 second denaturation at 98˚C, a 15 second 57˚C annealing, and a 30 second 72˚C extension with a final extension of 5 minutes at 72˚C, followed by a 4˚C hold. Note that primer designs for the 6p24.1 source element excluded the last 6 CpG sites captured in the Xp11.21 *FOXR2* insertion described below.

Bisulfite converted chrX-L1 DNA was amplified with the same above PCR reagents, except with a mixture of the following primers (600 nM ChrX-L1_bs_full-d2-For; 300 nM each ChrX-L1_bs_methyl-d2-Rev, ChrX-L1_vs_conv-site1-Rev, ChrX-L1_bs_conv-site2-Rev, ChrX-L1_bs_both-sites-Rev). The ChrX primers were designed to overlap the ORF2/5'UTR junction sequence of the FOXR2 L1 insertion. The thermocycling settings for amplification included an initial denaturation at 95˚C for 3 minutes, followed by 38 cycles of 98˚C denaturation for 20 seconds, 55˚C annealing for 15 seconds, and 72˚C extension for 30 seconds, and a final extension at 72˚C for 1 minute, followed by a 4˚C hold.

After PCR, primers and nucleotides were removed using a 1:1 ratio of Beckman Coulter Ampure beads A63880. Libraries were generated from the amplicons using the Roche Kapa Hyperprep DNA library kit. The amplicon library (4-6 nM) was loaded onto an Illumina MiSeq. Sequencing was performed using a 500-cycle v2 Nano reagent kit operating with v4 control software, and local run manager v3.0

Analysis of Bisulfite Converted Illumina MiSeq Fastq Reads

The generated 250 bp paird-end Miseq reads were trimmed using trimgalore v0.4.4 and cutadapt v1.8.1 with the default parameters to remove the adaptors.

For analysis of bisulfite sequence reads of the L1 5'UTR of the chr6p24.1 source element, trimmed MiSeq fastq reads were aligned with bismark v0.23.1dev [19] to the expected flanking genomic location and the 5'UTR of the chr6 amplified source element sequence [14](Suppl. Table S9D; element 6:13191033), requiring that all aligned reads align with no indels present. Passed reads were analyzed for presence of bisulfite converted nucleotides.

For analysis of the ORF2/5'UTR chrX *FOXR2* L1 sequence, the trimmed 250 bp paired-end reads were first merged into single-end reads based on overlap with bbmerge (bbmap, version 38.86, sourceforge.net/projects/bbmap/) set with parameter "forcemerge" to true [3]. Single-end reads ranging in size from 350 to 400 bp, the expected length of the inverted ORF2/5'UTR amplicon were aligned with bismark v0.23.1dev [19] to the expected sequence, requiring no indels in analyzed reads, and resulting reads displayed hypo-methylation.

Analysis of *FOXR2* Splicing Pattern in the Index HGG RNA-seq Samples

To confirm that *FOXR2* expression in the index HGG was caused by L1 promoter "donation" instead of L1 exonization, we generated a template sequence for the L1-inserted genomic region by inserting the ~3kb PacBio sequence outlined in Figure 1d to the L1 insertion site at chrX: 55,597,061 (GRCh38). We then map all RNA-seq reads to this L1-inserted template sequence by running STAR v2.7.9a in two-pass mode.

Reads aligned exclusively within the inserted L1 were removed as they could represent the expression of nearly identical L1 located in other genomic regions. For the same reason, reads

mapped within the repetitive regions of the template sequence were removed. Reads with >90% identity to the template sequence were retained for examining the splicing pattern.
The resulting RNA-seq read alignments are presented in Supplementary Fig. 2a using the IGV viewer. Somatic insertion of L1 5' UTR being the transcription initiation site can be verified by the following two patterns: 1) there is no splicing between the region upstream of the L1 insertion and the inserted cryptic L1, which excludes the possibility of L1 exonization; 2) all splice junction reads connecting to the acceptor site of *FOXR2* exon 2 were from the L1 5' UTR 97 donor sites; which shows that transcription only occurred on the L1-inserted mutant allele.

Detecting L1 Fusion Transcript Involving 97 Donor Site in Pediatric Brain Tumors RNA-seq
We used the L1.3 consensus sequence as the "bait" to identify chimeric reads with partial match to L1. Mapped RNA-seq bam files downloaded from the St. Jude Cloud were converted to fastq using Picard v2.6.0 and subsequently trimmed using trimgalore v0.6.7 and cutadapt v3.1 with the default parameters to remove the adaptors. The fastq files downloaded from EGAD00001001927 from the 22 previously published CNS tumors were trimmed as above [36] and underwent the same analysis as further. The trimmed fastq files were then mapped to the L1.3 consensus sequence [10] using bowtie2 v2.2.9 with "–very-sensitive-local parameter". Reads with edit distance ≤ 2, a threshold set to ensure we only retain reads likely belong to the young L1 elements similar to L1.3, were kept for future analysis.

To identify aberrant splicing events involving the L1 97 splice donor (SD) site, similar to the index HGG, we developed a custom script to extract the reads with a minimum overlap of 20 bp to the 97 SD, i.e. 78-97 bp of L1.3 in the remapped L1.3 bam file. For reads that pass this threshold and contain soft-clipped (SC) subsequences unaligned to L1.3, the soft-clipped reads

were then extracted and aligned to GRCh38 (GRCh38_no alt) to find the target exons by running bowtie2 v2.2.9 using the same parameters as above. Those that have ≥2 high-confidence match (<2 mismatches) to the reference genome were retained and annotated on the presence of L1 elements within 20 kb based on annotated polymorphic L1 on the Repeats Track: RepeatMasker of the UCSC genome browser (http://genome.ucsc.edu) [18] or somatic or germline L1 insertion identified by MELT v2.2.2 or Manta (details below).

For the 22 previously published CNS tumors we only assessed for presence of L1/FOXR2 fusion transcripts and unfortunately did not identify any and thus did not include these samples in Supplementary Table 2 nor Supplementary Table 3 results.

Identification of somatic L1 insertions using paired tumor-normal WGS data set

Manta v1.6.0 [4] was run with default settings on the paired WGS data and somatic insertions were manually inspected for bi-directional soft-clipped reads representing L1-related subsequence and poly-A tail. We confirmed that HGG WGS of our index sample can be detected by this approach. When using this approach to analyze the entire cohort, we identified a second event in a different HGG in an intergenic region on chromosome 7 (Supplementary Table 3).

MELT v2.2.2 [14] was run, first running the preprocess mode and then Melt-single mode only to L1 sequences as described at (https://melt.igs.umaryland.edu/manual.php) on tumor and matched normal samples independently. Events with a "PASS" filter were retained and further assessed manually for soft-clipped read support in IGV bam viewer for the presence of somatic L1 integration events. MELT did not identify the L1 insertion site in the index sample; however, it did discover two additional L1 insertion sites in our cohort (Supplementary Table 3).

TraFiC-mem was run through the docker container with default settings on the paired tumor-normal WGS data of our index case (GRCh37 BWA aligned) (https://gitlab.com/mobilegenomesgroup/TraFiC)[29] and no somatic L1 integration sites were identified. The results were also negative in the additional 3 HGG tumors, which had somatic L1 insertions detected by MELT and Manta (Supplementary Table 3) as well as the rest of the cohort with paired tumor-normal samples. GRCh37 BWA alignments were used for analysis as TraFiC-mem can only run on hg19 aligned samples.

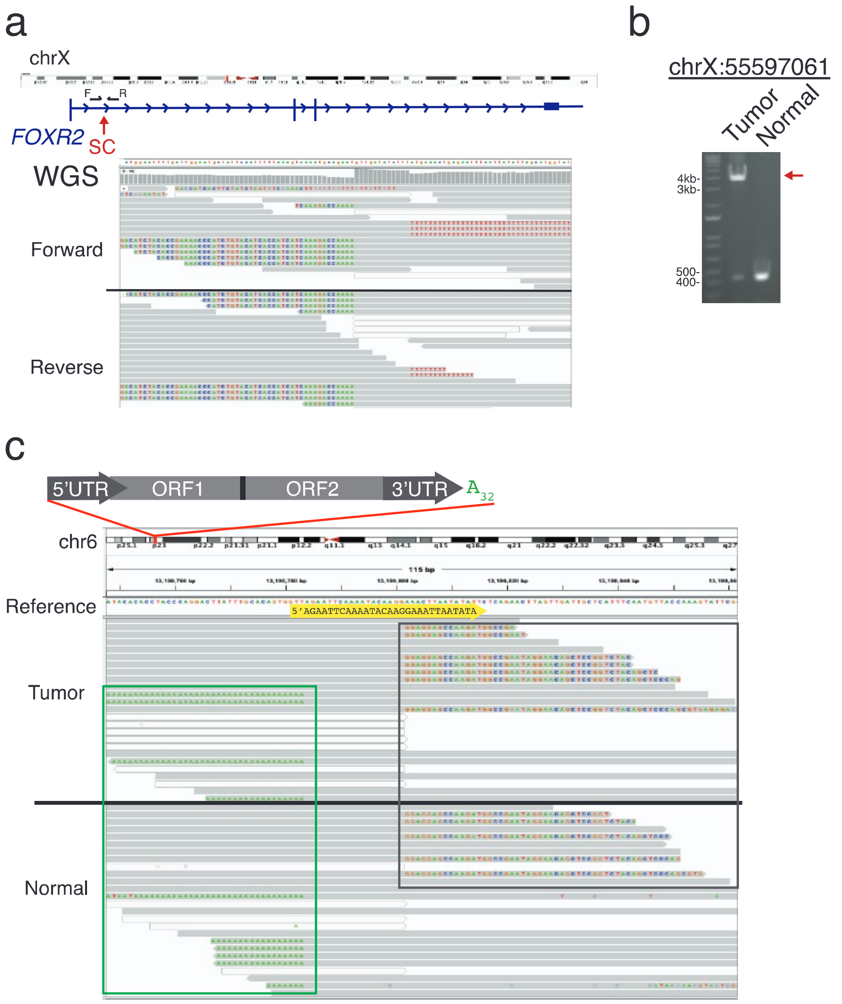

Supplementary Fig. 1. L1 Insertions in Index Patient a Schematic of non-canonical *FOXR2* isoform (blue) on the X chromosome (red vertical bar) with a cluster of soft-clipped reads (SC,

red arrow) observed in tumor whole genome sequencing below. Black arrows mark the forward (F) and reverse (R) primers used for PCR amplification results in supplementary Fig. 1b shown. **b** Amplification of the genomic region flanking the SC cluster at chrX:55597061 (GRCh38). The HGG displays a ~3.8kb amplicon (red arrow) while the matching germline sample a 423bp band as expected from the reference genome. Thermofisher's 1kb Plus ladder is shown on left. **c** WGS evidence of the 6p24.1 L1 source element in the tumor (top) and normal (bottom) with support of poly-A tail (green box) and L1 5'UTR (gray box).

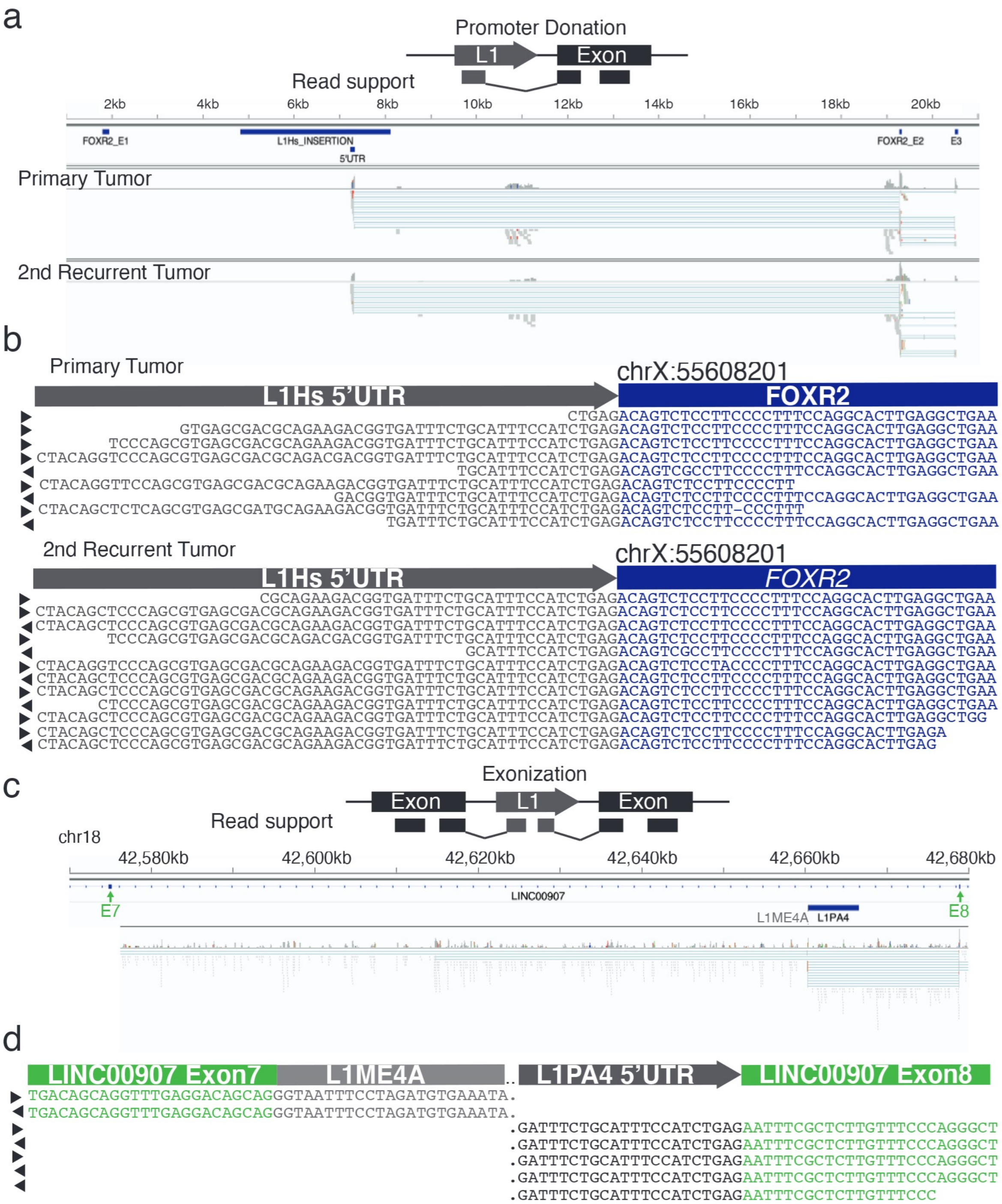

Supplementary Fig. 2. RNA Splicing Patterns a Splicing pattern in HGG RNA-seq supporting *FOXR2* activation via L1 promoter donation. Top, a schematic drawing of the promoter donation model where the inserted L1 serves as exon 1 for transcription initiation. Bottom, mapping of RNA-seq reads from the primary and 2nd recurrent HGG to a template sequence representing the mutant allele with somatic L1 insertion in the non-canonical intron 1 of *FOXR2*. The inserted cryptic L1 sequence which was determined by targeted PacBio sequencing shown in

Supplementary Table 1 is labeled as L1Hs_INSERTION. The absence of splicing from the genomic sequence upstream of the L1 insertion, which includes the non-canonical exon 1, to either the inserted L1 or the non-canonical exon 2 supports transcription initiation at L1 5' UTR (only first 97bp of L1 5'UTR are labeled). **b** Unique transcript reads supporting the splicing of L1 97 splice donor to *FOXR2* splice acceptor in the primary and 2nd recurrent tumor transcriptome. **c** An example of L1 exonization based on screening of 182 HGG RNA-seq. Top, schematic drawing of L1 exonization which involves splicing of an upstream exon to the inserted L1 site. Bottom, a L1 exonization example identified in RNA-seq data of SJHGG030665_D1 in the LINC00907 region where L1PA4 is integrated at intron 7 of LINC00907 as part of the reference human genome. RNA splicing was detected in the genomic region upstream of the L1PA4 integration site (*i.e.,* exon 7 and intron 7 in L1ME4A) to 5'UTR of L1PA4 in addition to splicing from L1PA4 to the downstream exon 8 of LINC00907, which matches the pattern of the L1 exonization model. **d** Unique transcript reads supporting the exonization example described in Supplementary Fig. 2c. Periods represent the additional genomic sequence present between the junctions shown.

a

	Primary	1st Recurrent	2nd Recurrent
WGS	N.T.	N.T.	YES
WES	YES	N.T.	YES
Transcriptome	YES	N.T.	YES
Methylation Array	YES	YES	YES

b

	Primary	1st Recurrent	2nd Recurrent
$TP53^{R175H}$ VAF	0	N.T.	0.9
$PDGFRA^{AMP}$	NO	NO	4-copy
*FOXR2/*L1	Yes	N.T.	Yes
t-sne Cluster	CNS NBL FOXR2	CNS NBL FOXR2	CNS NBL FOXR2

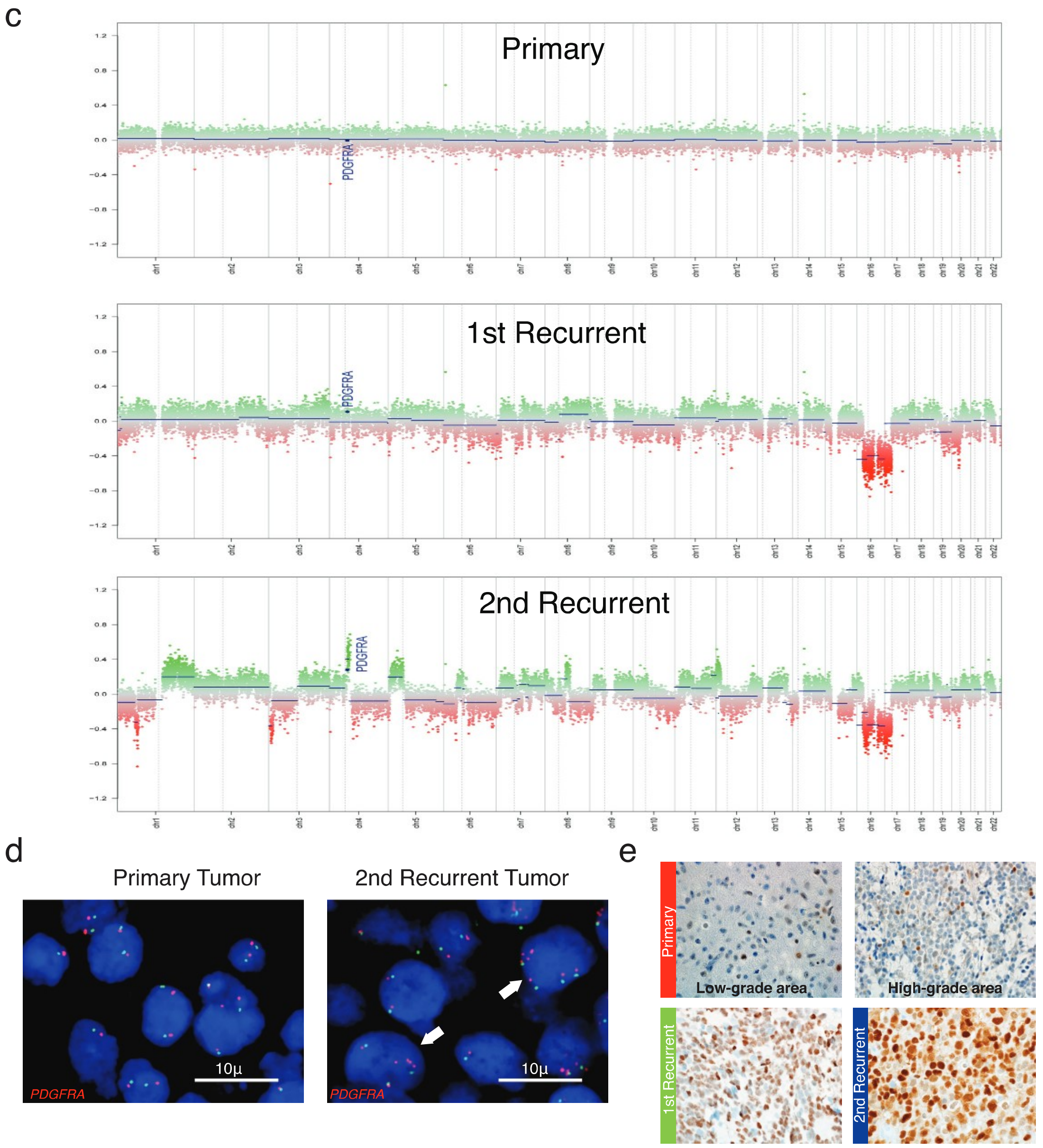

Supplementary Fig. 3. Molecular and genetic data of the HGG progression samples from the index patient a Summary table of molecular and genetic analysis done on respective

samples (N.T. means not tested). **b** Summary table of genetic variants discovered in the HGG progression samples. **c** Chromosomal copy number variations of the primary diagnosis sample (top), 1st recurrent tumor (middle), and 2nd recurrent tumor (bottom) analyzed by methylation array. A focal *PDGFRA* amplification was detected only at the 2nd recurrent tumor. **d** Fluorescence *in situ* hybridization (Red: *PDGFRA*, 4q12; Green: Control, 4p12) in the primary tumor (left) and 2nd recurrent tumor sample (right). White arrows indicate cells with *PDGFRA* amplification detected only at the 2nd recurrent tumor. **d** p53 IHC stains are shown for respective tumors.

Supplementary Table 1.
Sequence of flanking amplified *FOXR2* locus including the L1 insertion sequence and the subsequent matching SNPs to chr6p24.1 source element.

Supplementary Table 2.
Results of L1 97 splice donor transcript screening in 182 pediatric high grade glioma tumors.

Supplementary Table 3.
Additional intergenic somatic L1 insertions identified by Manta and MELT in the pediatric HGG cohort.

Supplementary Table 4.
Primer names and sequences used and described in materials and methods.

Supplementary Table 5.
A list of the pediatric high-grade glioma diagnosed sample ids, age of diagnosis, associated cohort, and type of sequencing data analyzed from the St. Jude Cloud.

References

1 Achanta P, Steranka JP, Tang Z, Rodic N, Sharma R, Yang WR, Ma S, Grivainis M, Huang CRL, Schneider AMet al (2016) Somatic retrotransposition is infrequent in glioblastomas. Mob DNA 7: 22 Doi 10.1186/s13100-016-0077-5

2 Belancio VP, Hedges DJ, Deininger P (2006) LINE-1 RNA splicing and influences on mammalian gene expression. Nucleic Acids Res 34: 1512-1521 Doi 10.1093/nar/gkl027

3 Bushnell B, Rood J, Singer E (2017) BBMerge - Accurate paired shotgun read merging via overlap. PLoS One 12: e0185056 Doi 10.1371/journal.pone.0185056

4 Chen X, Schulz-Trieglaff O, Shaw R, Barnes B, Schlesinger F, Kallberg M, Cox AJ, Kruglyak S, Saunders CT (2016) Manta: rapid detection of structural variants and indels for germline and cancer sequencing applications. Bioinformatics 32: 1220-1222 Doi 10.1093/bioinformatics/btv710

5 Chiang J, Diaz AK, Makepeace L, Li X, Han Y, Li Y, Klimo P, Jr., Boop FA, Baker SJ, Gajjar Aet al (2020) Clinical, imaging, and molecular analysis of pediatric pontine tumors lacking characteristic imaging features of DIPG. Acta Neuropathol Commun 8: 57 Doi 10.1186/s40478-020-00930-9

6 Chiang J, Harreld JH, Tinkle CL, Moreira DC, Li X, Acharya S, Qaddoumi I, Ellison DW (2019) A single-center study of the clinicopathologic correlates of gliomas with a MYB or MYBL1 alteration. Acta Neuropathol 138: 1091-1092 Doi 10.1007/s00401-019-02081-1

7 Chiang J, Li X, Liu APY, Qaddoumi I, Acharya S, Ellison DW (2020) Tectal glioma harbors high rates of KRAS G12R and concomitant KRAS and BRAF alterations. Acta Neuropathol 139: 601-602 Doi 10.1007/s00401-019-02112-x

8 Chiang JCH, Harreld JH, Tanaka R, Li X, Wen J, Zhang C, Boue DR, Rauch TM, Boyd JT, Chen Jet al (2019) Septal dysembryoplastic neuroepithelial tumor: a comprehensive clinical, imaging, histopathologic, and molecular analysis. Neuro Oncol 21: 800-808 Doi 10.1093/neuonc/noz037

9 Dobin A, Davis CA, Schlesinger F, Drenkow J, Zaleski C, Jha S, Batut P, Chaisson M, Gingeras TR (2013) STAR: ultrafast universal RNA-seq aligner. Bioinformatics 29: 15-21 Doi 10.1093/bioinformatics/bts635

10 Dombroski BA, Scott AF, Kazazian HH, Jr. (1993) Two additional potential retrotransposons isolated from a human L1 subfamily that contains an active retrotransposable element. Proc Natl Acad Sci U S A 90: 6513-6517 Doi 10.1073/pnas.90.14.6513

11 Downing JR, Wilson RK, Zhang J, Mardis ER, Pui CH, Ding L, Ley TJ, Evans WE (2012) The Pediatric Cancer Genome Project. Nat Genet 44: 619-622 Doi 10.1038/ng.2287

12 Flasch DA, Macia A, Sanchez L, Ljungman M, Heras SR, Garcia-Perez JL, Wilson TE, Moran JV (2019) Genome-wide de novo L1 Retrotransposition Connects Endonuclease Activity with Replication. Cell 177: 837-851 e828 Doi 10.1016/j.cell.2019.02.050

13 Fortin JP, Triche TJ, Jr., Hansen KD (2017) Preprocessing, normalization and integration of the Illumina HumanMethylationEPIC array with minfi. Bioinformatics 33: 558-560 Doi 10.1093/bioinformatics/btw691

14 Gardner EJ, Lam VK, Harris DN, Chuang NT, Scott EC, Pittard WS, Mills RE, Genomes Project C, Devine SE (2017) The Mobile Element Locator Tool (MELT): population-scale mobile element discovery and biology. Genome Res 27: 1916-1929 Doi 10.1101/gr.218032.116

15 He C, Xu K, Zhu X, Dunphy PS, Gudenas B, Lin W, Twarog N, Hover LD, Kwon CH, Kasper LHet al (2021) Patient-derived models recapitulate heterogeneity of molecular signatures and drug response in pediatric high-grade glioma. Nat Commun 12: 4089 Doi 10.1038/s41467-021-24168-8

16 Helman E, Lawrence MS, Stewart C, Sougnez C, Getz G, Meyerson M (2014) Somatic retrotransposition in human cancer revealed by whole-genome and exome sequencing. Genome Res 24: 1053-1063 Doi 10.1101/gr.163659.113

17 Keenan C, Graham RT, Harreld JH, Lucas JT, Jr., Finkelstein D, Wheeler D, Li X, Dalton J, Upadhyaya SA, Raimondi SCet al (2020) Infratentorial C11orf95-fused gliomas share histologic, immunophenotypic, and molecular characteristics of supratentorial RELA-fused ependymoma. Acta Neuropathol 140: 963-965 Doi 10.1007/s00401-020-02238-3

18 Kent WJ, Sugnet CW, Furey TS, Roskin KM, Pringle TH, Zahler AM, Haussler D (2002) The human genome browser at UCSC. Genome Res 12: 996-1006 Doi 10.1101/gr.229102

19 Krueger F, Andrews SR (2011) Bismark: a flexible aligner and methylation caller for Bisulfite-Seq applications. Bioinformatics 27: 1571-1572 Doi 10.1093/bioinformatics/btr167

20 Lee E, Iskow R, Yang L, Gokcumen O, Haseley P, Luquette LJ, 3rd, Lohr JG, Harris CC, Ding L, Wilson RKet al (2012) Landscape of somatic retrotransposition in human cancers. Science 337: 967-971 Doi 10.1126/science.1222077

21 Li B, Dewey CN (2011) RSEM: accurate transcript quantification from RNA-Seq data with or without a reference genome. BMC Bioinformatics 12: 323 Doi 10.1186/1471-2105-12-323

22 Li. H (2013) Aligning sequence reads, clone sequences and assembly contigs with BWA-MEM. arXiv 1:

23 Liu APY, Harreld JH, Jacola LM, Gero M, Acharya S, Ghazwani Y, Wu S, Li X, Klimo P, Jr., Gajjar Aet al (2018) Tectal glioma as a distinct diagnostic entity: a comprehensive clinical, imaging, histologic and molecular analysis. Acta Neuropathol Commun 6: 101 Doi 10.1186/s40478-018-0602-5

24 McLeod C, Gout AM, Zhou X, Thrasher A, Rahbarinia D, Brady SW, Macias M, Birch K, Finkelstein D, Sunny Jet al (2021) St. Jude Cloud: A Pediatric Cancer Genomic Data-Sharing Ecosystem. Cancer Discov 11: 1082-1099 Doi 10.1158/2159-8290.CD-20-1230

25 Miki Y, Nishisho I, Horii A, Miyoshi Y, Utsunomiya J, Kinzler KW, Vogelstein B, Nakamura Y (1992) Disruption of the APC gene by a retrotransposal insertion of L1 sequence in a colon cancer. Cancer Res 52: 643-645

26 Newman S, Nakitandwe J, Kesserwan CA, Azzato EM, Wheeler DA, Rusch M, Shurtleff S, Hedges DJ, Hamilton KV, Foy SGet al (2021) Genomes for Kids: The scope of pathogenic mutations in pediatric cancer revealed by comprehensive DNA and RNA sequencing. Cancer Discov: Doi 10.1158/2159-8290.CD-20-1631

27 Nguyen THM, Carreira PE, Sanchez-Luque FJ, Schauer SN, Fagg AC, Richardson SR, Davies CM, Jesuadian JS, Kempen MHC, Troskie RLet al (2018) L1 Retrotransposon

Heterogeneity in Ovarian Tumor Cell Evolution. Cell Rep 23: 3730-3740 Doi 10.1016/j.celrep.2018.05.090
28 Ritchie ME, Phipson B, Wu D, Hu Y, Law CW, Shi W, Smyth GK (2015) limma powers differential expression analyses for RNA-sequencing and microarray studies. Nucleic Acids Res 43: e47 Doi 10.1093/nar/gkv007
29 Rodriguez-Martin B, Alvarez EG, Baez-Ortega A, Zamora J, Supek F, Demeulemeester J, Santamarina M, Ju YS, Temes J, Garcia-Souto Det al (2020) Pan-cancer analysis of whole genomes identifies driver rearrangements promoted by LINE-1 retrotransposition. Nat Genet 52: 306-319 Doi 10.1038/s41588-019-0562-0
30 Rodriguez-Martin C, Cidre F, Fernandez-Teijeiro A, Gomez-Mariano G, de la Vega L, Ramos P, Zaballos A, Monzon S, Alonso J (2016) Familial retinoblastoma due to intronic LINE-1 insertion causes aberrant and noncanonical mRNA splicing of the RB1 gene. J Hum Genet 61: 463-466 Doi 10.1038/jhg.2015.173
31 Rusch M, Nakitandwe J, Shurtleff S, Newman S, Zhang Z, Edmonson MN, Parker M, Jiao Y, Ma X, Liu Yet al (2018) Clinical cancer genomic profiling by three-platform sequencing of whole genome, whole exome and transcriptome. Nat Commun 9: 3962 Doi 10.1038/s41467-018-06485-7
32 Samuelov L, Fuchs-Telem D, Sarig O, Sprecher E (2011) An exceptional mutational event leading to Chanarin-Dorfman syndrome in a large consanguineous family. Br J Dermatol 164: 1390-1392 Doi 10.1111/j.1365-2133.2011.10252.x
33 Scott EC, Devine SE (2017) The Role of Somatic L1 Retrotransposition in Human Cancers. Viruses 9: Doi 10.3390/v9060131
34 Scott EC, Gardner EJ, Masood A, Chuang NT, Vertino PM, Devine SE (2016) A hot L1 retrotransposon evades somatic repression and initiates human colorectal cancer. Genome Res 26: 745-755 Doi 10.1101/gr.201814.115
35 Shukla R, Upton KR, Munoz-Lopez M, Gerhardt DJ, Fisher ME, Nguyen T, Brennan PM, Baillie JK, Collino A, Ghisletti Set al (2013) Endogenous retrotransposition activates oncogenic pathways in hepatocellular carcinoma. Cell 153: 101-111 Doi 10.1016/j.cell.2013.02.032
36 Sturm D, Orr BA, Toprak UH, Hovestadt V, Jones DTW, Capper D, Sill M, Buchhalter I, Northcott PA, Leis Iet al (2016) New Brain Tumor Entities Emerge from Molecular Classification of CNS-PNETs. Cell 164: 1060-1072 Doi 10.1016/j.cell.2016.01.015
37 Tian Y, Morris TJ, Webster AP, Yang Z, Beck S, Feber A, Teschendorff AE (2017) ChAMP: updated methylation analysis pipeline for Illumina BeadChips. Bioinformatics 33: 3982-3984 Doi 10.1093/bioinformatics/btx513
38 Tubio JMC, Li Y, Ju YS, Martincorena I, Cooke SL, Tojo M, Gundem G, Pipinikas CP, Zamora J, Raine Ket al (2014) Mobile DNA in cancer. Extensive transduction of nonrepetitive DNA mediated by L1 retrotransposition in cancer genomes. Science 345: 1251343 Doi 10.1126/science.1251343
39 Wimmer K, Callens T, Wernstedt A, Messiaen L (2011) The NF1 gene contains hotspots for L1 endonuclease-dependent de novo insertion. PLoS Genet 7: e1002371 Doi 10.1371/journal.pgen.1002371
